# Molecular networks regulating cell division during Arabidopsis leaf growth

**DOI:** 10.1093/jxb/erz522

**Published:** 2019-12-07

**Authors:** Jasmien Vercruysse, Alexandra Baekelandt, Nathalie Gonzalez, Dirk Inzé

**Affiliations:** 1 Center for Plant Systems Biology, VIB, Gent, Belgium; 2 Department of Plant Biotechnology and Bioinformatics, Ghent University, Gent, Belgium; 3 INRAE, Université de Bordeaux, UMR1332 Biologie du fruit et Pathologie, INRA Bordeaux Aquitaine, Villenave d’Ornon cedex, France; 4 University of Antwerp, Belgium

**Keywords:** *Arabidopsis thaliana*, cell cycle, cell proliferation, leaf development, leaf size, organ growth

## Abstract

Leaves are the primary organs for photosynthesis, and as such have a pivotal role for plant growth and development. Leaf development is a multifactorial and dynamic process involving many genes that regulate size, shape, and differentiation. The processes that mainly drive leaf development are cell proliferation and cell expansion, and numerous genes have been identified that, when ectopically expressed or down-regulated, increase cell number and/or cell size during leaf growth. Many of the genes regulating cell proliferation are functionally interconnected and can be grouped into regulatory modules. Here, we review our current understanding of six important gene regulatory modules affecting cell proliferation during Arabidopsis leaf growth: ubiquitin receptor DA1–ENHANCER OF DA1 (EOD1), GROWTH REGULATING FACTOR (GRF)–GRF-INTERACTING FACTOR (GIF), SWITCH/SUCROSE NON-FERMENTING (SWI/SNF), gibberellin (GA)–DELLA, KLU, and PEAPOD (PPD). Furthermore, we discuss how post-mitotic cell expansion and these six modules regulating cell proliferation make up the final leaf size.

## Teaser

*Numerous* genes *have been identified that regulate leaf growth, which can be grouped into regulatory modules. Here, we review six important gene modules that affect cell proliferation during leaf development.*

## Introduction

Plants develop and grow mainly post-embryonically, forming two types of organs: organs such as roots with an indeterminate growth and therefore a theoretical unlimited growth potential; and organs such as leaves and flowers with a determinate growth and a fixed final size (Tsukaya, 2003; Rodriguez *et al.*, 2014). Leaves are the organs in which photosynthesis predominantly occurs. Leaves also contribute significantly to plant biomass, since the energy and carbohydrates produced during photosynthesis are used by the rest of the plant to sustain its growth and complete its life cycle (Demura and Ye, 2010). These features render leaf size control a highly interesting field of study.

In *Arabidopsis thaliana* (Arabidopsis), leaves grow through cell proliferation and cell expansion, two highly interconnected developmental processes, which are partially overlapping during leaf development (Asl *et al.*, 2011; Gonzalez *et al.*, 2012). Leaves are initiated by a group of founder cells emerging at the flanks of the shoot apical meristem (Reinhardt *et al.*, 2000; Efroni *et al.*, 2010; Kalve *et al.*, 2014). These leaf primordium founder cells undergo extensive cell division, resulting in an increased cell number that contributes to final leaf size (Gonzalez *et al.*, 2012). After a predefined developmental time frame, cells at the tip of the leaf exit the mitotic cell cycle and start to expand, marking the beginning of the cell expansion phase. In Arabidopsis, a cell cycle arrest front then moves through the leaf in a tip to base manner (Andriankaja *et al.*, 2012). However, some cells dispersed throughout the leaf epidermis retain their meristematic activity. These stem cell-like cells, called meristemoids, continue to divide asymmetrically for several rounds before giving rise to stomata, pores located in the epidermis that allow gas and water vapor exchange with the environment (Bergmann and Sack, 2007). In Arabidopsis, the increase in number of stomatal cells takes place in a tip to base direction as well, suggesting the occurrence of a secondary cell cycle arrest front corresponding to the arrest of meristemoid asymmetric divisions (White, 2006; Andriankaja *et al.*, 2012). Altogether, at least six major cellular processes contribute to final leaf size and shape: the number of cells recruited to the leaf primordium from the shoot apical meristem; the rate and duration of cell proliferation; the rate and duration of cell expansion; and the extent of meristemoid division (Gonzalez *et al.*, 2012; Hepworth and Lenhard, 2014). Impinging on one of these processes often results in an alteration in cell number or cell size, affecting final leaf size (Gonzalez *et al.*, 2012). Therefore, the correct regulation of cell proliferation and cell expansion mechanisms is fundamental to determine final leaf size.

**Fig. 1.**
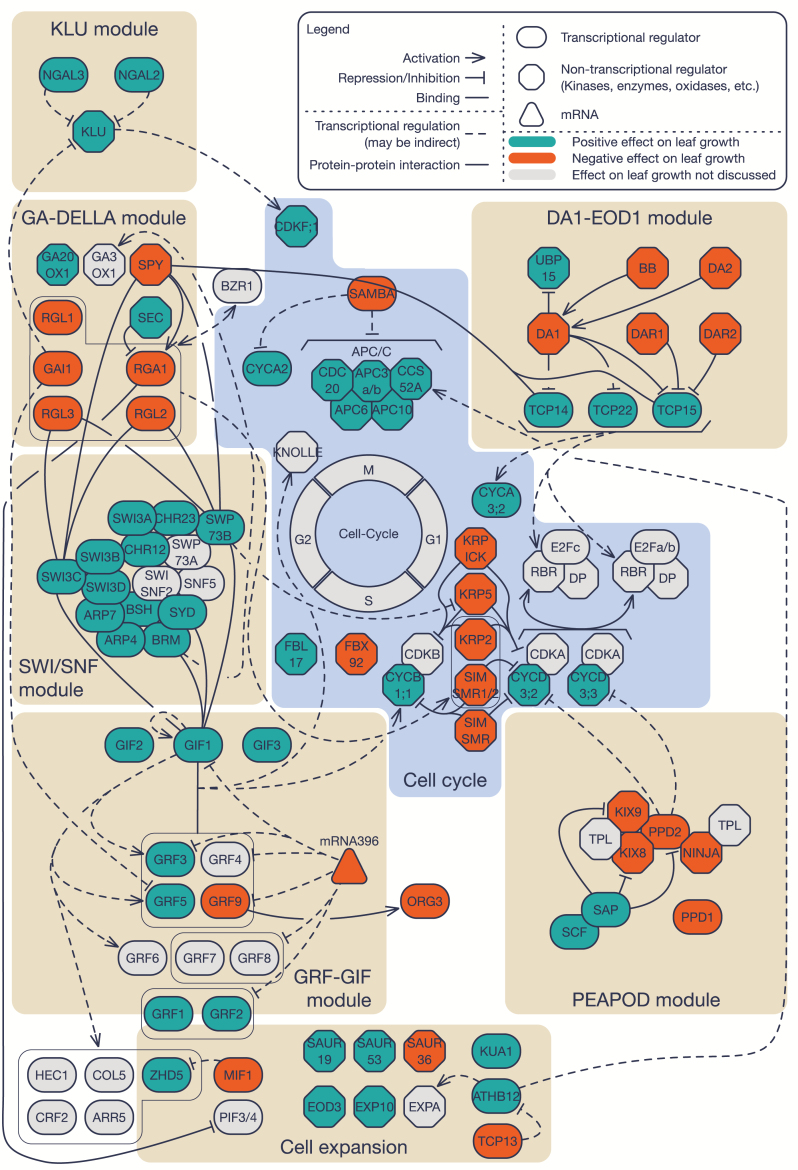
Overview of six of the gene regulatory modules known to be involved in cell proliferation and/or cell expansion: DA1–EOD1, GRF–GIF, SWI/SNF, GA–DELLA, KLU, and PEAPOD. The cell cycle is shown in the center and is surrounded by the core cell cycle proteins whose expression/activity is affected by one or more of the six regulatory modules. Proteins involved in cell expansion and their interaction with some of the modules are also indicated. Transcriptional (pill shapes) or non-transcriptional (octagonal shapes) regulators with a positive (teal blue) or negative (orange) effect on leaf growth are indicated. Gray proteins/transcriptional regulators are proteins whose effect on leaf growth is unknown or not presented in this review. The type of arrowhead indicates an activating (arrow) or repressing (T-junction) action, while absence of an arrowhead represents binding. These three actions are at either a transcriptional (dotted lines) or protein (solid lines) level. Abbreviations: *APC/C* (*ANAPHASE PROMOTING COMPLEX/CYCLOSOME*), *ARP* (*ACTIN RELATED PROTEINS*), *ARR* (*ARABIDOPSIS THALIANA RESPONSE REGULATOR*), *ATHB* (*ARABIDOPSIS THALIANA HOMEOBOX*), *BB* (*BIG BROTHER*), *BRM* (*BRAHMA*), *BSH* (*BUSHY*), *BZR* (*BRASSINAZOLE RESISTANT*), *CCS52A* (*CELL-CYCLE SWITCH PROTEIN*), *CDC20* (*CELL DIVISION CYCLE 20*), *CDK* (*CYCLIN DEPENDANT KINASE*), *COL5* (*CONSTANS-LIKE 5*), *CRF2* (*CYTOKININ RESPONSE FACTOR 2*), *CYC* (*CYCLIN*), *DAR* (*DA1-RELATED*), *DP* (*DIMERISATION PROTEIN*), *EOD* (*ENHANCER OF DA1*), *EXP* (*EXPANSIN*), *GA20OX1* (*GIBBERELLIN 20-OXIDASE 1*), *GA3OX1* (*GIBBERELLIN 3-OXIDASE 1*), *GAI1* (*GA INSENSITIVE*), *GIF* (*GRF-INTERACTING FACTOR*), *GRF* (*GROWTH REGULATING FACTOR*), *HEC1* (*HECATE 1*), *KIX* (*KINASE-INDUCIBLE DOMAIN INTERACTING*), *KRP/ICK* (*KIP-RELATED PROTEIN/INTERACTOR OF CDKs*), *KUA1* (*KUODA1*), *MIF1* (*MINI ZINC-FINGER 1*), *CHR* (*CHROMATIN REMODELING*), *NGAL* (*NGATHA-LIKE PROTEIN*), *NINJA* (*NOVEL INTERACTOR OF JAZ*), *ORG3* (*OBP3-RESPONSIVE GENE 3*), *PIF* (*PHYTOCHROME INTERACTING FACTOR*), *PPD* (*PEAPOD*), *RBR* (*RETINOBLASTOMA-RELATED*), *RGA1* (*REPRESSOR OF ga1-3*), *RGL* (*RGA-LIKE*), *SAP* (*STERILE APETALA*), *SAUR* (*SMALL AUXIN UP RNA*), *SCF* (*SKP1/CULLIN1/F-BOX PROTEIN*), *SEC* (*SECRET AGENT*), *SIM* (*SIAMESE*), *SMR* (*SIAMESE-RELATED*), *SNF5* (*SUCROSE NON-FERMENTING 5*), *SPY* (*SPINDLY*), *SWI/SNF* (*SWITCH/SUCROSE NON-FERMENTING*), *SWI3* (*SWITCH*), *SWP73* (*SWI/SNF ASSOCIATED PROTEIN 73*), *SYD* (*SPLAYED*), *TCP* (*TEOSINTE BRANCHED 1/CYCLOIDEA/PROLIFERATING CELL NUCLEAR ANTIGEN FACTOR*), *TPL* (*TOPLESS*), *UBP15* (*UBIQUITIN SPECIFIC PROTEASE 15*), *ZHD5* (*ZINC-FINGER HOMEODOMAIN 5*).

In this review, we describe current advances in Arabidopsis leaf growth regulation, mainly focusing on six gene regulatory modules involved in cell proliferation: DA1–ehhancer of DA1 (EOD1), GROWTH REGULATORY FACTOR (GRF)–GRF-INTERACTING FACOR (GIF), SWITCH/SUCROSE NON-FERMENTING (SWI/SNF), gibberellin (GA)–DELLA, KLU, and PEAPOD. We describe not only the components within each regulatory module, but also the connections between the modules, and how they are connected with the cell cycle, and, to a lesser extent, with the post-mitotic cell expansion machinery (Fig. 1; Table 1). The majority of the genes discussed throughout this review affect cell proliferation, demonstrating how the cell cycle and its machinery are central in mediating leaf growth. There are also many other aspects of leaf development including mechanisms controlling cell growth of dividing cells, such as leaf initiation (Ichihashi and Tsukaya, 2015; Sluis and Hake, 2015), leaf shape (Nikolov *et al.*, 2019; Sapala *et al.*, 2019), and the effect of environmental stress (Dubois *et al.*, 2018). Many of the processes are governed by plant hormones (Du *et al.*, 2018). However, to keep this review concise, emphasis is given to the regulation of cell division and to a lesser extent the contribution of post-mitotic cell expansion to leaf size.

**Table 1.** AT code, gene name, and description of genes discussed or mentioned in this review as well as the module to which they belong

Module	AT code	Gene name	Gene description
–	AT1G75080	BZR1	BRASSINAZOLE RESISTANT 1
–	AT1G75950	SKP1/ASK1/UIP1	S PHASE KINASE-ASSOCIATED PROTEIN 1/ARABIDOPSIS SKP1 HOMOLOGUE 1/UFO INTERACTING PROTEIN 1
–	AT3G48100	ARR5/IBC6	ARABIDOPSIS THALIANA RESPONSE REGULATOR 5/INDUCED BY CYTOKININ 6
–	AT3G56980	ORG3/BHLH039	OBF-BINDING PROTEIN 3 (OBP3)-RESPONSIVE GENE 3/BASIC HELIX-LOOP-HELIX 39
–	AT4G02570	CUL1/ICU13	CULLIN 1/INCURVATA 13
–	AT4G18710	BIN2	BRASSINOSTEROID-INSENSITIVE 2
–	AT4G23750	CRF2/TMO3	CYTOKININ RESPONSE FACTOR 2/TARGET OF MONOPTEROS 3
–	AT5G57660	COL5/BBX6	CONSTANS-LIKE 5/B-BOX DOMAIN PROTEIN 6
–	AT5G67060	HEC1	HECATE 1
Cell expansion	AT1G09530	PIF3	PHYTOCHROME INTERACTING FACTOR 3
Cell expansion	AT1G19840	SAUR53	SMALL AUXIN UPREGULATED RNA 53
Cell expansion	AT1G26770	EXP10	EXPANSIN 10
Cell expansion	AT1G74660	MIF1	MINI ZINC-FINGER 1
Cell expansion	AT1G75240	ZHD5/HB33	ZINC-FINGER HOMEODOMAIN 5/HOMEOBOX PROTEIN 33
Cell expansion	AT2G43010	PIF4	PHYTOCHROME INTERACTING FACTOR 4
Cell expansion	AT2G45210	SAUR36/SAG201	SMALL AUXIN UPREGULATED 36/SENESCENCE-ASSOCIATED GENE 201
Cell expansion	AT2G46660	EOD3/CYP78A6	ENHANCER OF DA-1 3/CYTOCHROME P450, FAMILY 78, SUBFAMILY A, POLYPEPTIDE 6
Cell expansion	AT3G02150	TCP13/PTF1	TEOSINTE BRANCHED 1/CYCLOIDEA/PROLIFERATING CELL NUCLEAR ANTIGEN FACTOR 13/PLASTID TRANSCRIPTION FACTOR 1
Cell expansion	AT3G61890	ATHB12	ARABIDOPSIS THALIANA HOMEOBOX 12
Cell expansion	AT5G18010	SAUR19	SMALL AUXIN UP RNA 19
Cell expansion	AT5G47390	KUA1/MYBH	KUODA1/MYB HYPOCOTYL ELONGATION-RELATED
Cell expansion	Gene group	PP2C	2C PROTEIN PHOSPHATASE
Cell expansion	Gene group	EXPA	EXPANSIN A
Cell cycle machinery	AT1G08560	KNOLLE/SYP111	SYNTAXIN OF PLANTS 111
Cell cycle machinery	AT1G32310	SAMBA	SAMBA
Cell cycle machinery	AT3G07870	FBX92	F-BOX PROTEIN 92
Cell cycle machinery	AT3G54650	FBL17	F-BOX LIKE 17
Cell cycle machinery	Gene group	APC/C	ANAPHASE PROMOTING COMPLEX/CYCLOSOME
Cell cycle machinery	Gene group	CCS52A	CELL CYCLE SWITCH PROTEIN 52 A
Cell cycle machinery	Gene group	CDC20	CELL DIVISION CYCLE 20
Cell cycle machinery	Gene group	CDK	CYCLIN DEPENDANT KINASE
Cell cycle machinery	Gene group	CYC	CYCLIN
Cell cycle machinery	Gene group	DP	DIMERISATION PROTEIN
Cell cycle machinery	Gene group	KRP/ICK	KIP-RELATED PROTEIN/INTERACTOR OF CDKs
Cell cycle machinery	Gene group	RBR	RETINOBLASTOMA-RELATED
Cell cycle machinery	Gene group	SIM	SIAMESE
Cell cycle machinery	Gene group	SMR	SIAMESE-RELATED
DA1–EOD1	AT1G14920	GAI/RGA2	GIBBERELLIC ACID INSENSITIVE/RESTORATION ON GROWTH ON AMMONIA 2
DA1–EOD1	AT1G15550	GA3OX1	GIBBERELLIN 3-OXIDASE 1
DA1–EOD1	AT1G17110	UBP15/SOD2	UBIQUITIN-SPECIFIC PROTEASE 15/SUPPRESSOR OF DA1 2
DA1–EOD1	AT1G19270	DA1	DA1
DA1–EOD1	AT1G69690	TCP15	TEOSINTE BRANCHED 1/CYCLOIDEA/PROLIFERATING CELL NUCLEAR ANTIGEN FACTOR 15
DA1–EOD1	AT1G72010	TCP22	TEOSINTE BRANCHED 1/CYCLOIDEA/PROLIFERATING CELL NUCLEAR ANTIGEN FACTOR 22
DA1–EOD1	AT1G78420	DA2	DA2
DA1–EOD1	AT2G39830	DAR2	DA1-RELATED PROTEIN 2
DA1–EOD1	AT3G47620	TCP14	TEOSINTE BRANCHED 1/CYCLOIDEA/PROLIFERATING CELL NUCLEAR ANTIGEN FACTOR 14
DA1–EOD1	AT3G63530	BB/EOD1	BIG BROTHER/ENHANCER1 OF DA1
DA1–EOD1	AT4G25420	GA20OX1	GIBBERELLIN 20-OXIDASE 1
DA1–EOD1	AT4G36860	DAR1	DA1-RELATED PROTEIN 1
GA–DELLA	AT1G66350	RGL1	RGA-LIKE 1
GA–DELLA	AT2G01570	RGA1	REPRESSOR OF GA1-3
GA–DELLA	AT3G03450	RGL2	RGA-LIKE 2
GA–DELLA	AT3G04240	SEC	SECRET AGENT
GA–DELLA	AT3G11540	SPY	SPINDLY
GA–DELLA	AT4G24210	SLY	SLEEPY 1
GA–DELLA	AT5G17490	RGL3	RGA-LIKE PROTEIN 3
GA–DELLA		GID2	GIBBERELLIN INSENSITIVE DWARF 2
GRF–GIF	AT1G01160	GIF2	GRF1-INTERACTING FACTOR 2
GRF–GIF	AT2G06200	GRF6	GROWTH-REGULATING FACTOR 6
GRF–GIF	AT2G22840	GRF1	GROWTH-REGULATING FACTOR 1
GRF–GIF	AT2G36400	GRF3	GROWTH-REGULATING FACTOR 3
GRF–GIF	AT2G45480	GRF9	GROWTH-REGULATING FACTOR 9
GRF–GIF	AT3G13960	GRF5	GROWTH-REGULATING FACTOR 5
GRF–GIF	AT3G52910	GRF4	GROWTH-REGULATING FACTOR 4
GRF–GIF	AT4G00850	GIF3	GRF1-INTERACTING FACTOR 3
GRF–GIF	AT4G24150	GRF8	GROWTH-REGULATING FACTOR 8
GRF–GIF	AT4G37740	GRF2	GROWTH-REGULATING FACTOR 2
GRF–GIF	AT5G28640	GIF1/AN3	GRF1-INTERACTING FACTOR 1/ANGUSTIFOLIA 3
GRF–GIF	AT5G53660	GRF7	GROWTH-REGULATING FACTOR 7
KLU	AT1G13710	KLU/CYP78A5	KLUH/CYTOCHROME P450, FAMILY 78, SUBFAMILY A, POLYPEPTIDE 5
KLU	AT3G11580	NGAL2/SOD7	NGATHA-LIKE PROTEIN 2/SUPRESSOR OF DA1 7
KLU	AT5G06250	NGAL3/DPA4	NGATHA-LIKE PROTEIN 3/DEVELOPMENT-RELATED PcG TARGET IN THE APEX 4
PPD	AT1G15750	TPL/WSIP1	TOPLESS/WUS-INTERACTING PROTEIN 1
PPD	AT3G24150	KIX8	KINASE-INDUCIBLE DOMAIN INTERACTING 8
PPD	AT4G14713	PPD1	PEAPOD 1
PPD	AT4G14720	PPD2	PEAPOD 2
PPD	AT4G28910	NINJA	NOVEL INTERACTOR OF JAZ
PPD	AT4G32295	KIX9	KINASE-INDUCIBLE DOMAIN INTERACTING 9
PPD	AT5G35770	SAP/SOD3	STERILE APETALA/SUPRESSOR OF DA1 3
SWI/SNF	AT1G18450	ARP4	ACTIN-RELATED PROTEIN 4
SWI/SNF	AT2G28290	SYD/CHR3	SPLAYED/CHROMATIN REMODELING COMPLEX SUBUNIT R 3
SWI/SNF	AT2G46020	BRM	BRAHMA
SWI/SNF	AT3G01890	SWP73A/CHC2	SWI/SNF ASSOCIATED PROTEIN 73 A
SWI/SNF	AT3G06010	CHR12	CHROMATIN REMODELING 12
SWI/SNF	AT3G17590	BSH	BUSHY GROWTH
SWI/SNF	AT3G17590	SNF5	SUCROSE NON-FERMENTING 5
SWI/SNF	AT3G60830	ARP7	ACTIN-RELATED PROTEIN 7
SWI/SNF	AT5G14170	SWP73B/CHC1	SWI/SNF ASSOCIATED PROTEIN 73 B
SWI/SNF	AT5G19310	CHR23	CHROMATIN REMODELING 23
SWI/SNF	Gene group	SWI/SNF	SWITCH/SUCROSE NON-FERMENTING
SWI/SNF	Gene group	SWI3	SWITCH

Protein groups or families represent multiple genes and therefore have no AT code.

## The pivotal role of the cell cycle machinery during leaf growth

During division, cells separate their duplicated genetic information into two daughter cells. This process, referred to as the cell cycle, can be subdivided into four phases: the S-phase during which the nuclear DNA is duplicated, the M-phase or mitosis during which the chromosomes are separated and distributed to the daughter cells, and two gap phases (G_1_ and G_2_) to prepare the cells for DNA replication or mitosis, respectively (Inzé and De Veylder, 2006). To ensure correct transmission of the genetic information, progression through these different phases is tightly controlled by different groups of core cell cycle proteins; the CYCLINS (CYCs) complexed with CYCLIN-DEPENDENT KINASES (CDKs), the E2F/DIMERISATION PROTEIN (DP) transcriptional regulatory proteins, KIP-RELATED PROTEIN/INTERACTOR OF CDKs (KRP/ICK), and SIAMESE/SIAMESE-RELATED (SIM/SMR) proteins (Inzé and De Veylder, 2006; Harashima *et al.*, 2013).

In Arabidopsis, CYCs include A-type CYCs (CYCA), B-type CYCs (CYCB), and D-type CYCs (CYCD), while CDKs include A-type CDKs (CDKA) and B-type CDKs (CDKB), the latter being plant specific (Vandepoele *et al.*, 2002; Inzé and De Veylder, 2006). The composition and activity of the CDK–CYC complexes are highly cell cycle phase specific, with CYCAs and CYCDs mainly involved in G_1_ progression and G_1_ to S transition, and CYCBs mainly regulating the G_2_ to M transition and progression through mitosis (Inzé and De Veylder, 2006; Van Leene *et al.*, 2011; Zhao *et al.*, 2012). In parallel, CDKAs are essential at both G_1_ to S and G_2_ to M phases, whereas CDKBs mainly control the G_2_ to M phase, progression through mitosis, and cell cycle exit (Inzé and De Veylder, 2006; Harashima *et al.*, 2013). The expression of genes required for G_1_ to S transition and S-phase progression is predominantly controlled by three E2F proteins (E2Fa, E2Fb, and E2Fc) that form heterodimeric complexes with DP proteins (DPa and DPb) (Magyar *et al.*, 2000; Kosugi and Ohashi, 2002; Desvoyes *et al.*, 2006; Yao *et al.*, 2018). While the E2Fc/DP complex is a transcriptional inhibitor, E2Fa/DP and E2Fb/DP complexes are transcriptional activators, whose activity is inhibited by binding to RETINOBLASTOMA-RELATED (RBR) proteins (Desvoyes *et al.*, 2006; Inzé and De Veylder, 2006). During the G_1_ to S transition, CYCD proteins are predominantly complexed with CDKA;1 (Boruc *et al.*, 2010; Van Leene *et al.*, 2011) that binds and phosphorylates RBR proteins associated with the E2Fa-b/DP complex, causing RBR degradation (Huntley *et al.*, 1998; Nakagami *et al.*, 1999; del Pozo *et al.*, 2006). The activated E2Fa-b/DP transcription factor complex triggers the expression of numerous target genes involved in cell cycle progression, transcription, chromatin dynamics, and DNA replication (Vandepoele *et al.*, 2005; Yao *et al.*, 2018). During transition between the G_2_- and M-phase, CDKA–CYCB complexes activate MYB3R proteins that in their turn activate several M-phase-related genes such as *KNOLLE* and *CYCB1;1* itself, guiding cell cycle exit into mitosis (De Veylder *et al.*, 2007). Alternatively, however, cells can continue to duplicate their genomic content (S-phase) for several rounds without subsequent division, called endoreduplication (Inzé and De Veylder, 2006; Breuer *et al.*, 2014).

The activity of the CDK–CYC complexes is tightly regulated by multiple mechanisms, acting at a transcriptional and a mainly post-translational level (Inzé and De Veylder, 2006; De Veylder *et al.*, 2007; Breuer *et al.*, 2014; Edgar *et al.*, 2014). These regulatory mechanisms include phosphorylation, interaction with cell cycle inhibitor proteins of the KRP/ICK and SIM/SMR family, and proteolysis mediated by the anaphase-promoting complex/cyclosome (APC/C) and the SKP1/CULLIN1/F-BOX PROTEIN (SCF) complexes (Inzé and De Veylder, 2006; Heyman and De Veylder, 2012). KRP/ICK proteins predominantly inhibit CDKA–CYCD complexes (Van Leene *et al.*, 2010). In lines overexpressing KRP proteins, mitosis is hampered, leading to a drastic decrease in cell number that is partially compensated by an increase in cell size (De Veylder et al., 2001, 2011). While single *krp* mutants do not show drastic effects, triple (*krp4/6/*7), quadruple (*krp1/2/6/7*), and quintuple (*krp1/2/5/6/7*) *krp* mutants have longer and enlarged leaves, which are narrow and curled downwards as a result of an increased cell number (Cheng *et al.*, 2013). A septuple *krp* mutant, in which all seven KRP/ICK genes are inactivated, produces leaves with an increased leaf size, similar to that in the quintuple *krp* mutant (Cao *et al.*, 2018). The SIM/SMR proteins inhibit CDKA–CYCD and CDKB–CYCB complexes, blocking progression through the cell cycle and promoting endoreduplication (Walker *et al.*, 2000; Churchman *et al.*, 2006; Van Leene *et al.*, 2010). Although *sim* mutants do not have an altered leaf phenotype, they have multicellular and clustered trichomes, and *SIM*-overexpressing plants are dramatically reduced in size (Walker *et al.*, 2000; Churchman *et al.*, 2006; Kumar *et al.*, 2015). The APC/C complex is a multiple subunit E3 ligase that controls cell cycle progression and endocycle entry, and altered expression levels of APC/C complex members, their activators, or their inhibitors impair plant morphology. APC10 is an essential component of the APC/C complex and, upon *APC10* overexpression, epidermal cells divide more quickly owing to a faster degradation of the mitotic cyclin CYCB1;1, resulting in the formation of enlarged leaves (Eloy *et al.*, 2011). Down-regulation of *APC10* or *APC6*, encoding another APC/C subunit, results in the production of smaller and curled leaves that show a reduced cell area (Marrocco *et al.*, 2009). In Arabidopsis, two isoforms exist for the APC/C subunit APC3: APC3a/CDC27a and APC3b/HOBBIT (Heyman and De Veylder, 2012). These proteins act with APC10 as receptors for the APC/C activators CELL CYCLE SWITCH PROTIEN 52 A/B (CCS52A/B) and CELL DIVISION CYCLE 20 (CDC20) (Fülöp *et al.*, 2005; Eloy *et al.*, 2011; Kevei *et al.*, 2011; Breuer *et al.*, 2012). Plants highly overexpressing *CCS52A* have a reduced leaf area as a result of a decreased cell number, slightly compensated by an increased cell area. Milder overexpression of *CCS52A*, however, results in larger plants because of increased cell divisions during the early stages of leaf development (Baloban *et al.*, 2013). Overexpression of *APC3a*/*CDC27a* increases leaf size owing to an increased cell number, whereas plants in which the expression of *APC3b/HOBBIT* is down-regulated are extremely dwarfed (Willemsen *et al.*, 1998; Heyman and De Veylder, 2012). APC/C is negatively regulated by SAMBA. Loss-of-function mutation in *SAMBA* (*samba*) results in plants that produce a larger shoot apical meristem, larger leaf primordia, and enlarged mature leaves, proposed to result, at least partially, from an increase in leaf primordium founder cells (Eloy *et al.*, 2012). SAMBA targets mitotic cyclins such as CYCLIN A2 (CYCA2) for APC/C-mediated degradation and eventually cell cycle exit (Eloy *et al.*, 2012). Accordingly, CYCA2s are stabilized in *samba* mutants throughout early leaf development, stimulating cell division (Eloy *et al.*, 2012).

F-box proteins are a major type of E3 ligases some of which are involved in cell cycle control, marking proteins for ubiquitin-mediated proteasomal degradation (Skaar *et al.*, 2013). Recently, it was described that overexpression of *F-BOX PROTEIN 92* (*AtFBX92*) results in the formation of smaller leaves as a result of a decreased cell number, though slightly compensated by an increased cell size (Baute *et al.*, 2017). Conversely, plants with a decreased expression of *AtFBX92* (*amiFBX92*) exhibited larger leaves, resulting from an increased cell division rate (Baute *et al.*, 2017). In addition, the F-box protein F-BOX LIKE 17 (FBL17) was characterized as a positive growth regulator, because *fbl17* mutants display a drastic reduction in leaf area due to a decrease in cell number compared with wild-type plants (Noir *et al.*, 2015).

## The DA1–EOD1 module

The DA1–EOD1 module has an important role in controlling leaf growth by regulating several key growth regulatory proteins in a post-translational manner. Plants with a dominant-negative point mutation in the gene encoding peptidase DA1 (*da1-1*) display enlarged leaves that contain more cells owing to a prolonged cell proliferation phase (Li *et al.*, 2008; Dong *et al.*, 2017; Vanhaeren *et al.*, 2017). In these plants, not only is the leaf area increased, but also the size of flowers, fruits, and seeds. In contrast, a decreased leaf size is observed upon overexpression of *GFP-DA1*, probably because DA1 is stabilized by the fluorescent tag, demonstrating that DA1 is a negative regulator of leaf growth (Vanhaeren *et al.*, 2017).

The peptidase activity of DA1 is activated upon multiple mono-ubiquitination by the E3 ligases BIG BROTHER/ENHANCER OF DA1 (BB/EOD1; referred to from hereon as BB) and DA2 (Xia *et al.*, 2013; Dong *et al.*, 2017). BB mutants (*bb-1*) exhibit smaller but shorter leaves, leaving total leaf area unchanged, and larger floral organs (Disch *et al.*, 2006). Overexpression of *BB* in the *bb-1* mutant background decreases leaf size drastically by restricting cell proliferation duration (Disch *et al.*, 2006). Plants in which *DA2* is mutated (*da2-1*) display larger leaves and have an increased biomass compared with the wild type, whereas overexpressing lines form smaller plants with a decreased leaf area (Xia *et al.*, 2013). While overexpression of *BB* or *DA2* dramatically decreases leaf size (Disch *et al.*, 2006; Xia *et al.*, 2013), *bb* and *da2* mutations in the *da1-1* mutant background synergistically enhance the *da1-1* phenotype (Li *et al.*, 2008; Xia *et al.*, 2013; Dong *et al.*, 2017; Vanhaeren *et al.*, 2017).

Several targets of DA1 have so far been described. Among others, DA1 negatively regulates the stability of the deubiquitinating enzyme SUPPRESSOR OF DA1 2/UBIQUITIN SPECIFIC PROTEASE 15 (SOD2/UBP15, referred to from hereon as UBP15) (Liu *et al.*, 2008; Du *et al.*, 2014; Dong *et al.*, 2017). Overexpression of *UBP15* leads to the formation of larger leaves, roots, flowers, and seeds as a result of increased cell divisions, mimicking the *da1-1* mutant phenotype (Liu *et al.*, 2008; Du *et al.*, 2014). In agreement with this, *ubp15-1* mutants have smaller organs compared with the wild type (Liu *et al.*, 2008; Du *et al.*, 2014), and the *da1-1* enlarged seed phenotype is repressed in *da1-1/ubp15* double mutants (Du *et al.*, 2014). In addition to UBP15, DA1 also inactivates TEOSINTE BRANCHED 1/CYCLOIDEA/PROLIFERATING CELL NUCLEAR ANTIGEN FACTOR 14 (TCP14), TCP15, and TCP22, transcription factors that positively regulate cell division duration (Dong *et al.*, 2017). More specifically, TCP14 and TCP15 repress the transition from mitosis to endoreduplication by inducing the expression of *RBR* and *CYCA3;2* (Li *et al.*, 2012; Peng *et al.*, 2015). The stability of TCP14 and TCP15 is modulated not only by DA1, but also by its close family members DA1-RELATED 1 (DAR1) and DAR2 (Peng *et al.*, 2015). Nonetheless, whereas the *da1-ko/dar1-1/dar2-1* triple mutant produces enlarged flowers and seeds, leaf size is decreased compared with wild-type plants, suggesting that DA1, DAR1, and DAR2 may regulate plant growth and development in an organ-specific manner (Peng *et al.*, 2015).

## The GRF–GIF module

The GRF–GIF module plays an important role in cell number determination in leaves. It consists of several interacting proteins of which ANGUSTIFOLIA3/GRF-INTERACTING FACTOR 1 (AN3/GIF1, referred to from hereon as GIF1) and members of the GROWTH REGULATING FACTOR (GRF) are transcriptional regulators (Kim and Kende, 2004; Debernardi *et al.*, 2014). The three GIF family members, GIF1, GIF2, and GIF3, are transcriptional co-activators that act, at least partially, redundantly to activate cell proliferation in the leaf primordia (Kim and Kende, 2004; Horiguchi *et al.*, 2005). Overexpression of *GIF1* results in plants that form enlarged organs resulting from an increased cell proliferation, reflected by an increased expression of *CYCB1;1* and other cell cycle-related genes (Lee *et al.*, 2009). In contrast, *gif1* mutants display smaller and narrower leaves that contain fewer cells compared with the wild type (Kim and Kende, 2004; Horiguchi *et al.*, 2005; Lee *et al.*, 2009). Accordingly, overexpression of *GIF2* and *GIF3* also increases leaf size by an increased cell number, demonstrating that GIF proteins act as positive regulators of cell proliferation (Lee *et al.*, 2009). Recently, it was shown that GIF1 might act as a mobile growth factor that diffuses through the leaf using plasmodesmata and, as such, establishes a long-range gradient along the leaf proximal–distal axis to determine the cell proliferation domain (Kawade *et al.*, 2017).

GIF1 was shown to interact with six out of the nine members comprising the GRF protein family in Arabidopsis: GRF1, GRF2, GRF3, GRF4, GRF5, and GRF9 (Kim and Kende, 2004; Horiguchi *et al.*, 2005; Debernardi *et al.*, 2014; Vercruyssen *et al.*, 2015). Overexpression of *GRF5* results in larger organs owing to an increased cell number, whereas down-regulation of *GRF5* results in the formation of narrower leaves that contain fewer cells (Horiguchi *et al.*, 2005; Kim and Tsukaya, 2015; Vercruyssen *et al.*, 2015). Several other members of the GRF family are also positive regulators of growth, such as GRF1 and GRF2, of which overexpression results in the formation of larger leaves (Kim and Tsukaya, 2015; Omidbakhshfard *et al.*, 2015). In contrast, however, GRF9 negatively regulates leaf growth, since overexpression of *GRF9* decreases organ size and the *grf9* mutant produces bigger leaf primordia, rosette leaves, and petals, resulting from an increased cell proliferation compared with wild-type plants (Omidbakhshfard *et al.*, 2018). GRF9 acts as a growth repressor by activating the expression of *OBF-BINDING PROTEIN 3* (*OBP3*)*-RESPONSIVE GENE 3* (*ORG3/bHLH039*, referred to from hereon as *ORG3*), which encodes a basic LEUCINE-ZIPPER (bZIP) transcription factor (Omidbakhshfard *et al.*, 2018). Whereas *org3* loss-of-function mutants produce leaves with an increased area as a result of an increased cell number compared with wild-type plants, the opposite phenotype is observed in plants overexpressing *ORG3* (Omidbakhshfard *et al.*, 2018). Consistent with the genetic interaction between *GRF9* and *ORG3*, the decreased leaf area in plants overexpressing *GRF9* (*GRF9ox*) is completely restored in *GRF9ox/org3* double mutants (Omidbakhshfard *et al.*, 2018). Several downstream target genes of GIF1 have been identified so far, including *GIF1* itself, *GRF3*, *GRF5*, *GRF6*, *TARGET OF MONOPTEROS 3/CYTOKININ RESPONSE FACTOR 2* (*TMO3/CRF2*), *B-BOX DOMAIN PROTEIN 6/CONSTANS-LIKE 5* (*BBX6*/*COL5*), *HECATE* (*HEC1*), *ZINC-FINGER HOMEODOMAIN 5/HOMEOBOX PROTEIN 33* (*ZHD5/HB33*, referred to from hereon as *ZHD5*), and *ARABIDOSPIS THALIANA RESPONSE REGULATOR 5* (*ARR5*) (Vercruyssen *et al.*, 2014).

Except for GRF5 and GRF6, GRF family members are regulated at the transcript level by miR396-mediated RNA cleavage (Liu *et al.*, 2009; Rodriguez *et al.*, 2010; Debernardi *et al.*, 2014). *miR396* expression increases throughout leaf development in a basipetal direction, following the cell cycle arrest front, restricting *GRF* expression to the basal part of the leaf (Liu *et al.*, 2009; Rodriguez *et al.*, 2010; Wang *et al.*, 2011). Since the balance between *GRF* genes and *miR396* regulates cell number in a quantitative manner, *miR396*-overexpressing plants produce small and narrow leaves containing fewer cells owing to a shorter cell proliferation phase (Liu *et al.*, 2009; Rodriguez *et al.*, 2010; Wang *et al.*, 2011). In contrast, overexpression of a *miR396-*resistant version of *GRF3* (*rGRF3*) prolongs cell proliferation, resulting in the formation of larger leaves that contain more cells (Debernardi *et al.*, 2014).

## The SWI/SNF chromatin remodeling module

The SWITCH/SUCROSE NON-FERMENTING (SWI/SNF) chromatin remodeling complex is closely linked with the GRF–GIF module and can activate and/or repress transcription by disrupting DNA–histone interactions, thereby altering chromatin accessibility (Han *et al.*, 2015; Archacki *et al.*, 2017). The SWI/SNF complex comprises a functional core including a SWI2/SNF2 ATPase family member, BRAHMA (BRM), SPLAYED (SYD), CHROMATIN REMODELING 12 (CHR12) or CHR23 (Han *et al.*, 2015), an SNF5 subunit, BUSHY (BSH), two SWI/SNF ASSOCIATED PROTEINS 73 (SWP73A/CHC2 and SWP73B), two ACTIN RELATED PROTEINS (ARP4 and ARP7), and a pair of SWI3 subunits: SWI3A, SWI3B, SWI3C, or SWI3D (Vercruyssen *et al.*, 2014). SWI/SNF subunits are important for transcriptional regulation of key developmental processes (Wagner and Meyerowitz, 2002; Farrona *et al.*, 2004; Hurtado *et al.*, 2006; Kwon *et al.*, 2006). Loss of function in the double knockout *CHR12/CHR23* (*minu1/minu2*), *SWI3A*, *SWI3B*, or *ARP7* causes embryonic lethality. Whereas plants with a single mutation in *BRM*, *SYD*, *SWI3C*, or *SWI3D* or showing silencing of *BSH*, *SWP73B*, or *ARP4* do manage to develop, they display severe embryonal defects with limited leaf and flower development, often resulting in sterility (Kandasamy *et al.*, 2005a, b; Sarnowski *et al.*, 2005; Sang *et al.*, 2012; Sacharowski *et al.*, 2015). The *brm* mutant exhibits pleiotropic phenotypic alterations, resulting in an overall reduced plant size accompanied by a downward curling of the leaves (Farrona *et al.*, 2004; Hurtado *et al.*, 2006; Tang *et al.*, 2008). Furthermore, overexpression of *SWI3C* enhances leaf growth by increasing the number of cells (Vercruyssen *et al.*, 2014), whereas *swi3c* mutants display small rosettes constituted of curled leaves (Sacharowski *et al.*, 2015). GIF1 associates with the SWI/SNF complex through several subunits, including BRM, SYD, and SWP73B, to induce transcription of several downstream cell cycle-related genes (Vercruyssen *et al.*, 2014).

## The GA–DELLA module

Gibberellins (GAs) play an important role in both cell proliferation and cell expansion, and mutations in genes involved in GA signaling or homeostasis can drastically affect plant organ size (Achard *et al.*, 2009). Overexpression of *GIBBERELLIN 20-OXIDASE 1* (*GA20OX1*), encoding a rate-limiting enzyme essential for GA biosynthesis, results in increased levels of active GA, leading to the formation of enlarged leaves that contain more and larger cells (Coles *et al.*, 1999; Gonzalez *et al.*, 2010). In contrast, plants with reduced GA levels or a reduced GA sensitivity display a dwarfed phenotype (Olszewski *et al.*, 2002). In Arabidopsis, there are five DELLA proteins; GA INSENSITIVE (GAI), REPRESSOR OF *ga1-3* (RGA), RGA-LIKE 1 (RGL1), RGL2, and RGL3. All five DELLA proteins function as key repressors of GA-responsive growth, inhibiting GA-regulated gene expression (Sun and Gubler, 2004; de Lucas *et al.*, 2008). GA binds to the GIBBERELLIN INSENSITIVE DWARF 2 (GID2) receptor, which causes ubiquitination of the DELLA proteins, marking them for protein degradation with the help of F-box protein SLEEPY 1 (SLY1) and the SCF^SLY1/GID2^ E3 ligase complex (McGinnis *et al.*, 2003; Dill *et al.*, 2004; Ueguchi-Tanaka *et al.*, 2007). Plants in which DELLA proteins are stabilized (*sleepy1*), which are GA deficient (*ga1-3*), or in which *SLY* is mutated (*sly1-10*), show a dwarfed phenotype (Olszewski *et al.*, 2002; Dill *et al.*, 2004; Fu *et al.*, 2004). In contrast, the quadruple DELLA mutant (*gai-t6/rga-t2/rgl1-1/rgl2-1*), mimicking constitutive GA signaling, displays increased cell division rates, and consequently larger leaves (Achard *et al.*, 2009).

To regulate transcription, DELLA proteins exert their inhibiting function through protein–protein interactions with other transcriptional regulators (de Lucas *et al.*, 2008). Among others, RGA is known to interact with and inhibit the transcriptional activity of PHYTOCRHOME INTERACTING FACTOR 3 (PIF3) and PIF4, basic HELIX-LOOP-HELIX (bHLH) factors involved in light signaling and mediators of cell elongation (de Lucas *et al.*, 2008). Further downstream, DELLA proteins promote the expression of the cell cycle inhibitor-encoding genes *KRP2*, *SIM*, *SMR1*, and *SMR2, because* the expression of these cell cycle genes is elevated in GA-deficient plants, suggesting that the resulting dwarfed phenotype is caused by inhibition of the cell cycle (Achard *et al.*, 2009). In addition to their involvement in the GA pathway, DELLA proteins are linked to the brassinosteroid pathway, since they regulate and are regulated by BRASSINAZOLE RESISTANT 1 (BZR1), which in its turn is inhibited by BRASSINOSTEROID INSENSITIVE 2 (BIN2), known to positively affect cell proliferation. Furthermore, DELLA proteins are regulated through protein modification by SECRET AGENT (SEC) and SPINDLY (SPY) (Zentella *et al.*, 2017). SEC acts as a positive growth regulator by inducing a closed conformation of the DELLA protein RGA1 through the addition of *O*-β-*N*-acetylglucosamine, inhibiting the repressor activity of RGA1 (Zentella *et al.*, 2017). The loss-of-function mutant *sec-2* displays a reduction in leaf length compared with wild-type plants (Hartweck *et al.*, 2006). In contrast, SPY acts as a negative regulator of growth by enhancing the capacity of RGA1 to bind to PIF3, PIF4, and BZR1 (Zentella *et al.*, 2017). Reduced SPY activity partially suppresses the dwarfed phenotype caused by *ga1* that lacks an early GA biosynthesis enzyme (Filardo and Swain, 2003). In contrast, mutations in *SEC* do not reverse the dwarfed phenotypes in a *ga1* background, demonstrating that its role might be GA signaling specific (Hartweck *et al.*, 2006).

## The KLU module

KLU/KLUH/CYP78A5 (referred to from hereon as KLU) is a plant-specific cytochrome P450 protein belonging to the CYP78A subfamily. The CYP78A subfamily consists of six members in Arabidopsis termed CYP78A5–CYP78A10, and stimulates cell proliferation during leaf, flower, seed, and fruit development (Anastasiou *et al.*, 2007; Adamski *et al.*, 2009; Eriksson *et al.*, 2010). It is proposed that KLU stimulates cell proliferation in a non-cell-autonomous manner, either by producing a mobile growth-promoting molecule or by degrading a, so far unknown, growth-inhibiting signal (Anastasiou *et al.*, 2007; Eriksson *et al.*, 2010). Loss of KLU function also shortens the time between successive leaf initiation events, referred to as the plastochron, leading to an increased final leaf number (Wang *et al.*, 2008). Accordingly, *KLU* is expressed at the boundary between the shoot apical meristem and developing organ primordia, further strengthening its putative role in leaf initiation (Zondlo and Irish, 1999). KLU is proposed to stimulate cell proliferation, at least to some extent, redundantly with the closely related protein CYP78A7, because the loss-of-function *cyp78a7* mutant does not show a clear phenotype, whereas seedlings of *cyp78a5/cyp78a7* double mutants are smaller compared with wild-type plants (Wang *et al.*, 2008).

The expression of *KLU* is repressed by SUPPRESSOR OF DA1-1 7/NGATHA-LIKE PROTEIN 2 (SOD7/NGAL2), a B3 transcription factor that binds directly to the *KLU* promoter (Zhang *et al.*, 2015). Accordingly, the smaller leaf phenotype in the dominant *sod7-1D* mutant may directly result from an increased expression of *KLU*, though largely unexplored so far. The closest homolog of NGAL2, DEVELOPMENT-RELATED PcG TARGET IN THE APEX4 (DPA4)/NGAL3, also seems to regulate plant size since, in the absence of DPA4/NGAL3, leaves appear smaller as a result of a decreased cell number compared with the wild type (Zhang *et al.*, 2015). Additionally, KLU is regulated by the DELLA protein GAI1, which may link the KLU module with the GA–DELLA module, although this is largely underexplored so far (Claeys *et al.*, 2014).

## The PEAPOD module

In the epidermis of Arabidopsis leaves, 48% of the pavement cells are estimated to originate from the repeating asymmetric divisions of meristemoids, stem cell-like precursor cells of the stomatal lineage (Larkin *et al.*, 1997; Geisler *et al.*, 2000). Consequently, the extent of meristemoid division also contributes significantly to final leaf size (White, 2006; Gonzalez *et al.*, 2015). Meristemoid asymmetric division is negatively regulated by PEAPOD 1 (PPD1) and PPD2, putative DNA-binding proteins that belong to the TIFY protein family, a plant-specific group of proteins with a broad range of functions (White, 2006; Zhang *et al.*, 2012; Gonzalez *et al.*, 2015). Landsberg *erecta* (L*er*) plants in which the *PPD* locus is deleted (∆*ppd*) and Col-0 plants expressing an artificial miRNA targeting the *PPD* transcripts (*ami-ppd*) both form enlarged rosettes with enlarged dome-shaped leaves that contain more cells owing to an increased meristemoid division compared with wild-type leaves (White, 2006; Gonzalez *et al.*, 2015). In contrast, overexpression of the *PPD* genes results in the formation of leaves that are smaller and flatter, containing fewer cells compared with wild-type leaves (White, 2006).

PPD proteins interact with KINASE-INDUCIBLE DOMAIN INTERACTING 8 (KIX8), KIX9 and NOVEL INTERACTOR OF JAZ (NINJA), acting as adaptor proteins for the co-repressor TOPLESS (TPL) (Gonzalez *et al.*, 2015; Baekelandt *et al.*, 2018). The *kix8/kix9* double mutant phenocopies both the *ami-ppd* leaf size and shape, suggesting that KIX8 and KIX9 act in a redundant manner and are pivotal for PPD functionality (Gonzalez *et al.*, 2015). Whereas *ninja* mutants also show dome-shaped leaves, they lack the leaf size increase observed in *ami-ppd* and *kix8/kix9* plants (Baekelandt *et al.*, 2018). PPD2 is known to bind to the promoters of two out of the three *D3-type CYCLIN* genes, *CYCD3;2* and *CYCD3;3*, repressing their transcription, and, accordingly, the expression of *CYCD3;2* and *CYCD3;3* is increased in *ami-ppd*, *kix8/kix9*, and *ninja* leaves compared with the wild type (Gonzalez *et al.*, 2015; Baekelandt *et al.*, 2018). Interestingly, meristemoid initiation and activity are reduced in the *cycd3;1/cycd3;2/cycd3;3* triple mutant compared with the wild type (Dewitte *et al.*, 2007; Elsner *et al.*, 2012; Lau *et al.*, 2014). More recently, it has been shown that plants overexpressing *CYCD3;2* display propeller-like rosettes with narrow dome-shaped leaves, though lacking the leaf size increase observed in *ppd* and *kix8/kix9* mutants (Baekelandt *et al.*, 2018). Down-regulation of the expression of two out of the three *CYCD3* genes, *CYCD3;1* and *CYCD3;2*, can partially complement the *ami-ppd* leaf curvature phenotype, suggesting that *CYCD3* genes are direct PPD2 target genes involved in controlling leaf shape (Baekelandt *et al.*, 2018). In contrast, overexpression of *CYCD3;3* does not affect leaf shape, but results in an overall reduced growth, indicative that, though considered to act redundantly, CYCD3 proteins may have specific functions during leaf shape control (Baekelandt *et al.*, 2018).

In Arabidopsis, the activity of the PPD/KIX complex is regulated by the SCF complex containing the F-box protein STERILE APETALA/SUPPRESSOR OF DA1 3 (SAP/SOD3, referred to from hereon as SAP) (Wang *et al.*, 2016; Li *et al.*, 2018). Polyubiquitination of the PPD/KIX complex by SCF^SAP^ results in proteasome-dependent degradation of the protein complex (Wang *et al.*, 2016; Li *et al.*, 2018). Consistently, Arabidopsis plants overexpressing *SAP* produce enlarged leaves with uneven lamina growth and have an increased expression of the PPD/KIX downstream target genes *CYCD3;2* and *CYCD3;3* compared with wild-type plants (Wang *et al.*, 2016; Li *et al.*, 2018).

## Connecting the growth regulatory modules with the cell cycle

During recent years, more and more studies demonstrate that the six growth regulatory modules discussed here do not operate independently, and several links between the different modules and with the core cell cycle machinery have already been discussed (Fig. 1). DA1-mediated proteolysis of TCP14/15/22 results in the induction of *CYCA3;2* and *RBR* expression, whereas the PPD module regulates *CYCD3;2* and *CYCD3;3* expression (Baekelandt *et al.*, 2018), demonstrating that both modules regulate the G_1_/S transition of the cell cycle. Furthermore, the SWP73B subunit of the SWI/SNF complex is known to bind to the promoter of *KRP5*, encoding a cell cycle inhibitor that regulates endoreduplication and interacts with D-type CYCs, thereby also regulating the G_1_/S transition (Jégu *et al.*, 2013). Also the downstream target genes of the GRF transcription factors include many cell cycle-related genes, such as *KNOLLE*, which is active during the M-phase, when cell plate formation occurs (Lauber *et al.*, 1997; Touihri *et al.*, 2011), and *CYCB1;1*, pivotal for the G_2_/M transition (Debernardi *et al.*, 2014; Vercruyssen *et al.*, 2014). Additionally, inducible *KLU* overexpression in the *klu-2* mutant background causes up-regulation of *CDKF;1*, a CDK-ACTIVATING KINASE (CAK) affecting the activity of the CDK/CYC complexes throughout the cell cycle by phosphorylation (Umeda *et al.*, 2005; Takatsuka *et al.*, 2009). Plants lacking functional CDKF;1 exhibit a dwarfed phenotype because of a decreased cell number and cell size (Takatsuka *et al.*, 2009). Finally, DELLA proteins activate the expression of several genes encoding cell cycle inhibitors, such as KRP2, SIM, SMR1, and SMR2, that are responsible for the onset of endoreduplication and as such contribute to the balance between cell proliferation and endoreduplication during leaf development (Achard *et al.*, 2009; Kumar *et al.*, 2015).

In addition to the direct connections with the cell cycle, several interactions between the members of different regulatory modules have been described. The SWI/SNF and the GA–DELLA modules are directly connected through SWI3C, a subunit of the SWI/SNF complex, that interacts with the DELLA proteins RGL2 and RGL3, and the DELLA regulatory protein SPY (Sarnowska *et al.*, 2013). Furthermore, SPY is known to physically interact with TCP14 and TCP15, which are degraded in a DA1-dependent manner and repressed by DELLA proteins, connecting the SWI/SNF, GA–DELLA, and DA1–EOD1 modules (Steiner *et al.*, 2012; Davière *et al.*, 2014; Resentini *et al.*, 2015). Additionally, the BRM subunit was found to bind to the promoters of *GA3ox1* (Sarnowska *et al.*, 2013; Archacki *et al.*, 2016), affecting GA biosynthesis. The GRF–GIF and SWI/SNF modules are also closely connected, because GIF1 associates with the SWI/SNF complex through several subunits, including BRM, SYD, and SWP73B, to induce the expression of the downstream target genes (Vercruyssen *et al.*, 2014). Finally, upon expression of an inducible non-degradable form of GAI in proliferating leaf cells, *GRF5* and *KLU* transcripts are decreased, putatively linking the GA–DELLA, KLU, and GRF–GIF modules (Claeys *et al.*, 2014).

Phenotypic effects observed upon misexpression of individual members of distinct modules may also be balanced at the leaf level. For instance, whereas the DA1–EOD1 module predominantly affects the primary arrest front, the PPD module is mainly involved in establishing the secondary arrest front (Gonzalez *et al.*, 2012). Taken together, both are involved in determining cell proliferation, and therefore cell number and final leaf size. In agreement, at least two *SOD* mutants were identified in forward genetic screens that could so far not be directly linked with the DA1–EOD1 module: SAP that is part of the PPD module and NGAL2 that is part of the KLU module (Zhang *et al.*, 2015; Wang *et al.*, 2016). In both cases, it seems that the *da1-1* phenotype can be complemented by affecting distinct core cell cycle genes or impinging on different processes of leaf development.

## The importance of post-mitotic cell expansion for leaf growth

Besides cell proliferation, cell expansion contributes significantly to final leaf size, and a close coordination between cell proliferation and cell expansion is fundamental for proper organogenesis (Andriankaja *et al.*, 2012). Cell expansion is proposed to be predominantly regulated by EXPANSINs (EXPs), XYLOGLUCAN ENDOTRANSGLUCOSEYLASE/HYDROLASEs (XTHs), PECTIN METHYLESTERASEs (PMEs), and reactive oxygen species (ROS) (Cosgrove, 2015; Schmidt *et al.*, 2016). Auxin-induced acidification of the apoplast by ATPases importing H^+^ ions results in the activation of cell wall-associated EXPs that facilitate cell wall loosening (Cosgrove, 2000, 2005). Plants ectopically expressing *EXP10* display larger leaves and longer petioles containing larger cells, whereas down-regulation of *EXP10* has the inverse effect (Cosgrove, 2015). Also, SMALL AUXIN UP RNA (SAUR)-type proteins are proposed to promote ATPase activity by inhibiting 2C protein phosphatase (PP2C) proteins, resulting in the acidification of the apoplast and stimulating cell expansion (Chae *et al.*, 2012; Hou *et al.*, 2013). Plants ectopically expressing green fluorescent protein (GFP)-stabilized SAUR19 protein display an increased leaf area owing to the production of larger cells (Spartz et al., 2012, 2014). In contrast, *saur36* mutants produce bigger leaves containing larger cells, demonstrating that SAUR36 acts as a negative regulator of cell expansion (Hou *et al.*, 2013). Furthermore, SAUR53 has also been identified to positively regulate cell elongation, because ectopic expression of *SAUR53* results in the elongation of cells and organs (Kathare *et al.*, 2018). Another link between auxin and cell expansion was demonstrated by Katano *et al.* (2016). They showed that in *fugu5* mutants, lacking the *AVP1*-encoded H^+^-pyrophosphatase, cell division is inhibited, thus triggering auxin-induced compensated cell expansion (Katano *et al.*, 2016).

Besides EXP10 and several members of the SAUR family, only few other proteins have been described to impinge on the cell expansion phase, including GRF1, GRF2, EOD3/CYP78A6, ZHD5, KUODA 1 (KUA1), and ARABIDOPSIS THALIANA HOMEOBOX 12 (ATHB12) (Hong *et al.*, 2011; Fang *et al.*, 2012; Lu *et al.*, 2014; Hur *et al.*, 2015; Omidbakhshfard *et al.*, 2015; Tsukaya, 2015). In contrast to the increased cell numbers in plants overexpressing *GRF5* or *GRF9*, the increased leaf area in *GRF1*- and *GRF2*-overexpressing plants results from an increased cell area (Lee *et al.*, 2009; Omidbakhshfard *et al.*, 2015). Also in plants overexpressing *EOD3*, encoding a cytochrome P450 similar to KLU, seeds and leaves are bigger as a result of increased cell expansion, whereas *EOD3* down-regulation leads to smaller leaves that consist of smaller cells (Fang *et al.*, 2012). Also the transcriptional regulators ZHD5, KUA1, and ATHB12 positively regulate leaf growth, and their overexpression results in larger leaves and seeds owing to an increased cell area compared with the wild type (Hong *et al.*, 2011; Fang *et al.*, 2012; Lu *et al.*, 2014; Hur *et al.*, 2015). ZHD5 is part of the zinc-finger homeodomain (ZF-HD) class of transcription factors, which comprises 14 members in Arabidopsis that can homo- and heterodimerize (Tan and Irish, 2006; Hu *et al.*, 2008). ZHD5 activity can be abolished by MINI ZINC-FINGER 1 (MIF1), which also contains a zinc-finger domain but lacks a DNA-binding domain (Hu and Ma, 2006; Hong *et al.*, 2011). In this way, MIF1 acts as a competitive inhibitor peptide and, upon overexpression, blocks binding of ZHD5 to the DNA, resulting in dwarfed plants (Hu and Ma, 2006; Hong *et al.*, 2011). *KUA1* encodes a MYB-like transcription factor that positively regulates leaf growth by promoting cell wall relaxation (Lu *et al.*, 2014; Schmidt *et al.*, 2016). ATHB12 is involved in cell expansion as well as ploidy determination, since overexpression of *ATHB12* induces the expression of *CCS52A* and *CCS52B*, encoding components of the APC/C complex, regulating endoreduplication onset, as well as the expression of *EXPA*, involved in cell expansion (Hur *et al.*, 2015). Recently, TCP13 was found to repress *ATHB12* expression, and overexpression of *TCP13* results in a decreased leaf length and size owing to a reduction in cell size (Hur *et al.*, 2019). Similarly, down-regulation of *TCP13* and its paralogs, *TCP5* and *TCP17*, results in enlarged leaf cells, suggesting that TCP13 regulates cell expansion through transcriptional control of *ATHB12* (Hur *et al.*, 2019).

The alterations in organ size in mutants with an impaired cell division are often not as pronounced as one would expect based on the reduction in cell numbers (Ferjani *et al.*, 2007; Horiguchi and Tsukaya, 2011). This is because inhibition of cell division in organs with determinate growth, such as leaves, is often compensated by excessive post-mitotic cell expansion, a phenomenon called compensation (Hisanaga *et al.*, 2015). Interestingly, such compensatory mechanisms often occur in mutants of core cell cycle genes (Blomme *et al.*, 2014). For instance, the decreased cell number in the triple *cycd3* mutant is compensated by an increased cell area (Dewitte *et al.*, 2007). Also, *gif1* mutants and plants overexpressing *KRP2* show only a slight decrease in leaf area, because the decrease in cell number is partially restored by an increase in cell size (Mizukami and Fischer, 2000; De Veylder *et al.*, 2001; Horiguchi *et al.*, 2005; Kawade *et al.*, 2010). Analogously, the increased cell number in plants that ectopically express *E2Fa* is partially restored by a decreased cell size, resulting in the formation of slightly enlarged cotyledons and leaves (De Veylder *et al.*, 2002). Altogether, these findings strengthen the putative presence of complex interactions between cell division and cell expansion, coordinated by distinct mechanisms (Ferjani *et al.*, 2007; Horiguchi and Tsukaya, 2011). In this way, inhibition of one process may, at least partially, be restored by an increased activity of another process to ensure that the genetically determined size is attained as well as possible (Horiguchi *et al.*, 2006; Horiguchi and Tsukaya, 2011; Hisanaga *et al.*, 2015). The underlying molecular mechanisms, however, are often still largely underexplored (Ferjani *et al.*, 2007; Horiguchi and Tsukaya, 2011; Hisanaga *et al.*, 2015).

## Concluding remarks

In this review, we presented six modules that are important for Arabidopsis leaf size determination and showed that for most of them, direct links with the cell cycle machinery have been revealed. In addition, we demonstrate that connections between these different modules are revealed with an increasing pace. This demonstrates that the modules described throughout this review do not stand on their own, but that leaf growth is an intricate process that requires the cooperation of various interconnected key players that are part of complex regulatory networks. In the future, additional work will be required to further complete our view on these regulatory networks and the connections residing therein. There are also many genes affecting leaf size that were not presented in this review, largely because there are, to our knowledge, so far no links with any of the modules discussed here. In the future, more research will be required to also map these regulators in the bigger network of leaf growth regulation. Ultimately, mathematical modeling may enable us to fully grasp the complexity of the organ growth machinery.
